# Potential Influence of Myokines on Skeletal Muscle Tissue Hypertrophy Signaling Pathways: A Narrative Review

**DOI:** 10.3390/biom16060850

**Published:** 2026-06-10

**Authors:** Stephen M. Cornish, Jose Peralta-Huertas

**Affiliations:** 1 Applied Health Sciences Program, Faculty of Graduate and Postdoctoral Studies, University of Manitoba, Winnipeg, MB R3T 2N2, Canada; peraltaj@myumanitoba.ca; 2 Faculty of Kinesiology and Recreation Management, University of Manitoba, Winnipeg, MB R3T 2N2, Canada; 3 Centre for Aging, University of Manitoba, Winnipeg, MB R3T 2N2, Canada

**Keywords:** aging, anabolism, cytokine, hypertrophy, myokine, skeletal muscle

## Abstract

Sarcopenia is defined as the age-related loss of skeletal muscle strength, power, and size. Understanding the fundamental mechanisms whereby sarcopenia occurs is an area of research that has received much attention due to the aging population. Skeletal muscle tissue is used for locomotion and acts as a major site aiding the regulation of metabolism. Myokines are cytokines released from skeletal muscle tissue that act in an autocrine, paracrine, or endocrine manner. Myokines have been termed the ‘exercise factor’ or ‘work factor’ that scientists have long thought communicate between skeletal muscle and various physiological systems, including muscle-to-muscle cross-talk. One area of research that has been underexplored is the effect that myokines may have in an autocrine manner on skeletal muscle tissue itself. Although the myokine role in skeletal muscle hypertrophy and atrophy has been somewhat elucidated in rodent models, relatively little research has been performed in human models to understand the role myokines have on anabolic and catabolic metabolism in an autocrine manner. This review will provide an overview of myokine function within a biological context, some molecular pathways involved in skeletal muscle anabolism, a mechanistic understanding of myokine autocrine action, key evidence in relation to skeletal muscle satellite cell interaction with myokines, how myokines may be involved in skeletal muscle regeneration, and an outline of some key myokines that have the potential to act in an anabolic fashion within skeletal muscle. The review will then emphasize some important areas of research that are needed to understand the role of myokines in maintaining or improving skeletal muscle mass within an aging context.

## 1. Introduction

While the loss of skeletal muscle mass and function with age (sarcopenia) is an ever-present challenge for modern science and society [1], the underlying mechanisms whereby skeletal muscle hypertrophy and atrophy occur require more investigation to determine interventions that will best serve to preserve skeletal muscle into older age [2]. Dietary, pharmacological, and exercise interventions have shown potential promise in reducing the atrophic effects of age on skeletal muscle; however, there is still much to be gleaned from study in this area to understand how aged muscle differs from younger muscle in its response to stimuli that would promote anabolic responses. The presence of anabolic resistance, an attenuated response of skeletal muscle to anabolic stimuli in older age, is a perplexing problem which has not been completely solved with scientific endeavors [3]. Mechanistically understanding the fundamental molecular processes of how skeletal muscle adapts to stimuli in younger and older age may help researchers determine how to best reduce the risk of sarcopenia. There has been a heavy emphasis in the research literature on understanding the molecular pathways of skeletal muscle anabolism to aid in reducing the incidence of muscle loss with age [4]. However, there is a niche of research literature examining the potential for myokines, peptides, or proteins that are released from skeletal muscle and act in an autocrine, paracrine, or endocrine manner, to influence anabolic pathways in skeletal muscle. Our research group [5] and others [6,7,8] have reviewed this topic previously, but recent evidence points to the probable effect that myokines have in influencing skeletal muscle hypertrophy and the cross-talk between skeletal muscle and the immune system in regulating this process [9]. Thus, the purpose of this review is to provide relevant details pertaining to the effects that several key myokines (apelin, decorin, interleukin-1 receptor antagonist [IL-1ra], IL-6, IL-10, IL-15, irisin, and leukemia inhibitory factor [LIF]) have in influencing skeletal muscle hypertrophic responses and to highlight the need for more extensive research in this area.

## 2. Myokines

Myokines are proteins and peptides, many of which are also cytokines, released from skeletal muscle tissue which influence the myocyte directly (autocrine), other myocytes or cell types in proximity (paracrine), and other tissue types at a distance (endocrine). While the term myokine was coined over 20 years ago [10], there has been some contention in the literature as to where myokines originate, as it has been argued there are multiple cell types found within skeletal muscle that may be producing the myokine response systemically [11]. Myokine autocrine action on skeletal muscle is an underexplored area of research; however, some recent evidence indicates certain myokines may influence skeletal muscle regeneration, at least in vitro [12], and that interleukin-6 (IL-6) has a role in activating the mammalian target of the rapamycin complex-1 (mTORC1) pathway [13]. Myokine research has moved forward slowly and still requires further investigation to determine the exact role each myokine may play in skeletal muscle metabolism [6]. While the role of myokines has been somewhat elucidated in in vitro and rodent research models, much is left to be discovered regarding these small biomolecules in human research.

There are both pro- and anti-inflammatory myokines that are necessary for maintaining physiological homeostasis which may influence catabolism and anabolism, respectively. If the balance between the two main types of myokines is altered, the consequence could reduce skeletal muscle anabolism and enhance catabolism, producing undesirable outcomes on muscle mass and function [14]. In a naturally aged mouse model, resistance-exercise training promoted a decrease in a pro-inflammatory environment by stimulating M2 macrophage polarization; decreasing mRNA for tumor necrosis factor-α (TNF-α), interleukin-1β (IL-1β), and nuclear factor kappa-light-chain-enhancer of activated B-cells (NF-κB); and increasing IL-6 and interleukin-10 (IL-10) mRNA, which would promote an anti-inflammatory milieu supporting anabolic metabolism in skeletal muscle [15]. In support of this, a rat model found that recombinant IL-10 delivery versus saline modulated the immune response and improved volumetric muscle loss after skeletal muscle injury [16]. Further, treating male rats with infliximab (an anti-TNF-α biological pharmaceutical) versus saline after an induced skeletal muscle injury resulted in greater hypertrophy in the infliximab-treated group; however, there are likely key times in the repair and regeneration process whereby infliximab could be more or less beneficial [17]. Overall, myokines have an influence on skeletal muscle tissue, but the extent of that influence and the underlying mechanisms at work require further clarification.

## 3. Overview of Pathways Involved in Skeletal Muscle Protein Synthesis

The mammalian target of the rapamycin (mTOR) pathway is a main pathway whereby protein synthesis occurs in skeletal muscle [18]. In the mTOR pathway, there are a number of upstream and downstream regulators that control the synthesis of protein in skeletal muscle. Predominantly, the upstream initiation of the pathway occurs through insulin-like growth factor-1 (IGF-1) activating its receptor, which then phosphorylates insulin receptor substrate-1 (IRS-1). This leads to a number of signaling events that eventually phosphorylate protein kinase B (AKT), which then phosphorylates mTOR and ribosomal protein S6 kinase beta-1 (S6K1), leading to protein synthesis (for an up-to-date review on this, see [18]). Interestingly, phospholipase D (PLD) enhances mTOR signaling in the absence of IGF-1/Akt signaling and may be a compensatory mechanism that activates protein synthesis in skeletal muscle, at least in vitro [19,20]. There are, of course, other pathways involved in skeletal muscle protein degradation that will not be expanded on in this review; however, as previously mentioned, an excellent recent review on the topic can be found in [18].

## 4. Mechanistic Autocrine Actions of Myokines

The concept of myokines acting as autocrine signaling agents is somewhat difficult to study in vivo in skeletal muscle since most of these compounds have been identified in muscle biopsy homogenates after one bout of acute exercise or chronic exercise training. The myokines can be excreted by other cells found within the skeletal muscle homogenate such as leukocytes, pericytes, and fibroblasts [11,21,22,23]. Elevated concentrations of myokines in skeletal muscle only suggest the possibility that they are produced by skeletal muscle cells after physical exertion [22]. Due to this assumption, several compounds, including myokines, that seem to originate in skeletal muscle after acute or chronic exercise have been referred to as exerkines [22,23,24]. Several studies illustrate this: IL-6 is mainly localized in myofibers but also in satellite cells and fibroblasts [25,26]; LIF is found in endothelial cells [25]; while vascular endothelial growth factor (VEGF) is detected in myofibers, endothelial cells, and pericytes [27]. This heterogeneity of sources and locations of potential myokines is a confounding factor in this field [11]. Furthermore, most of the studies on myokines have been in rodents and not in humans [24]. It is common practice to use the term “myokine” irrespective of the validation of myofibers as the origin of the released biomolecule.

If autocrine action is defined as a signaling molecule that originates in the same cell that it is affecting, myokines may be one example of this physiological process in a number of metabolic pathways [21]. Some researchers consider skeletal muscle as an autocrine, paracrine, and endocrine organ able to produce hormone-like effects [22,28]. Myostatin acts as a catabolic compound in skeletal muscle [21,29]; IL-15 and LIF act as anabolic agents [22]; while others, like IL-10, enhance the microenvironment for muscular repair after injury by promoting the anti-inflammatory response following muscle-damaging exercise [30,31]. An increase in IL-10 leads to a transition from M1 phenotypic macrophages to M2 macrophages, which in turn start the production of IGF-1, support satellite cell differentiation, and promote extracellular matrix remodeling [30,31,32]. In some instances, certain myokines may act in tissues simultaneously, as in the case of neurturin, which initiates signaling pathways in both motor neurons and skeletal muscle [24]; meanwhile, others, like S100B and Semaphorin 3A, may do the same [33]. There is also evidence of metabolic regulation and fuel oxidization involving myokines illustrated by irisin, IL-6, and apelin actions. Increased irisin concentrations during exercise enhance glucose uptake within muscle fibers after exercise [34]; IL-6 increases insulin sensitivity by increasing glucose uptake during skeletal muscle action [13]; and apelin stimulates glucose uptake, increases insulin sensitivity, and regulates lipolysis and fatty acid oxidation [35], suggesting an important role as therapeutic tool against diseases like type II diabetes. Furthermore, BDNF provokes mitochondrial fission and clearance in skeletal muscle, controlling mitochondrial quality in murine specimens [36], while chronically combining BDNF with endurance training increased aerobic capacity in mice [37].

In relation to the potential autocrine role of myokines in skeletal muscle, a focus on recent research demonstrates a probable role for certain myokines to act in this manner. Irisin has been a myokine of interest for its role in skeletal muscle metabolism. Recent research in C2C12 myotubes indicates that irisin was able to ameliorate glucocorticoid-induced atrophy via inhibition of the forkhead box O (FoxO) 3α atrophic pathway, which was partially due to IGF-1 dependent signaling [38]. However, other in vivo research in rats has demonstrated that overexpression of fibronectin type III domain-containing 5 (Fndc5)/irisin did not enhance muscle protein synthesis [39]. Conversely, the sphingosine-1-phosphate/sphingosine-1-phosphate receptor (a biolipid mediator) seems to play a role in initiating Fndc5 formation, which eventually leads to irisin formation, promoting myoblast proliferation and differentiation in C2C12 myoblasts [40]. Interestingly, platelet-derived growth factor subunit B (PDGF-B) was constitutively expressed in myotubes and may act on myotubes and myoblasts as an autocrine myokine; however, when myotubes were contracted electrically for 60 min, there was not any increase in PDGF-B in the culture media or its expression within the myotubes [12]. Furthermore, an activation of the PI3K/Akt/mTOR pathway is required to stimulate FGF-21 production for its potential role as an autocrine/paracrine myokine in isolated muscle fibers from the flexor digitorum brevis in male BALB/c mice [41]. Further, FGF-21 is released into the systemic circulation with various types of exercise in humans [42] and is therefore a probable pleiotropic myokine that acts in an autocrine, paracrine, and endocrine manner. Finally, recent work that investigated the autocrine role of C1q/TNF-related protein 9 (CTRP9) in C2C12 myoblasts found that this myokine is required for sustaining myogenesis in aged myoblasts and that its absence impairs myogenic differentiation [43]. For a short summary of potential autocrine roles of myokines, please see Table 1.

## 5. Skeletal Muscle Satellite Cells

Skeletal muscle satellite cells (SC) respond to exercise stimuli and play a role in regeneration of skeletal muscle tissue following damage [61]. Skeletal muscle hypertrophy is influenced by having a net positive balance in muscle protein synthesis as well as the activation of SC accumulation to muscle fibers [62]. However, aging [63], biological sex [64], disease [23], as well as metabolism and nutritional influences [65] may all impact SC sequential events. Various growth factors including fibroblast growth factors (FGFs), transforming growth factor-β (TGF-β), and especially hepatocyte growth factor (HGF) heavily affect the activation of the SC cycle or return them to the quiescent state [66]. Mechanical stretch triggers the release of nitric oxide (NO), which acts as the primary stimulus for the activation and proliferation of skeletal muscle satellite cells (SC) [67]. These activated cells then express myomaker and myomerger to fuse with existing muscle fibers, providing the nuclear addition necessary for sustainable growth [68]. Most exerkines preserve or promote activation, differentiation, self-renewal, division, and proliferation of muscle satellite cells [23,69]; however, a few cytokines, specifically adinonectin and leptin, induce quiescence in SC [23]. A balanced transition between quiescence and activation of SC is essential for muscle homeostasis [70]. Activation of SC into myoblasts or a return to quiescence depends on physiological or pathological stimuli such as exercise, inflammation, injury, or sarcopenia [71], with the constant renewal of cells maintaining homeostasis. A very interesting approach highlighting the primordial importance of SC in muscular regeneration and application in aging-related states has been mentioned in the literature. Methods based on biochemical or genetic inhibition of aberrantly regulated intrinsic satellite cell signal transduction pathways and strategies regulating extrinsic factors have successfully achieved rejuvenation of aged SC [72]. This result points to the potential for SC to be manipulated, especially during the activation stage of their sequential events, to conserve regenerative ability even into old age [73]. Essentially, certain myokines such as irisin, IL-6, and BDNF have been shown to be intricately involved in the sequential events of SC and the attenuation of skeletal muscle atrophy [23]. However, due to changing systemic and tissue concentrations of various myokines with age, these myokines may alter the physiological processes and produce pathological effects on skeletal muscle tissue [74].

## 6. Postnatal Myogenesis (Muscle Regeneration)

Postnatal skeletal muscle myogenesis is a repair system that maintains skeletal muscle integrity after birth. Postnatal myogenesis relies on satellite cells to respond to the specific demands of injury and exercise in an established organism [75]. Skeletal muscle regeneration is a sequential process that includes the following satellite cell phases: quiescence, activation, migration, myogenic determination, myoblast proliferation, myocyte differentiation, myofiber maturation, and hypertrophy [75]. The skeletal muscle cell may go from a dormant state to a hypertrophic phase with an increase in cell size and protein content. It includes a few trigger stimuli to activate cells and migrate to the pathological/damaged area, to differentiate into specific lineage, and to clone into many new cells, developing into mature myoblasts, fusing into structural strands, and growing to support the whole functional ability of skeletal muscle [75].

**Figure 1:**
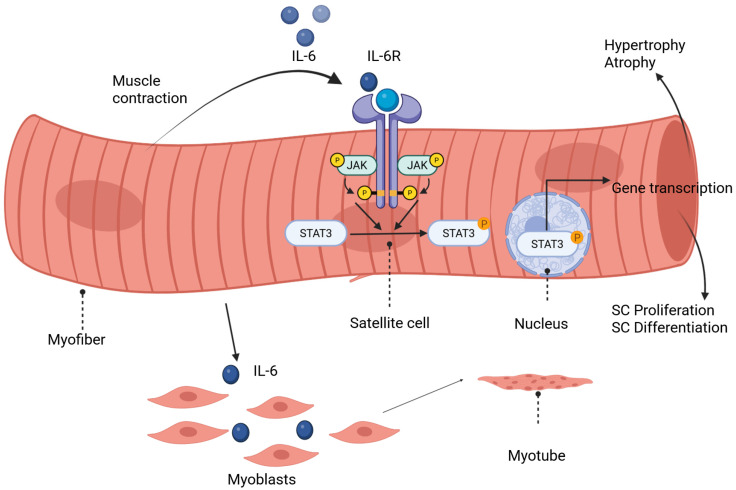
An example of the potential role of interleukin-6 (IL-6) in signaling gp130 receptor containing Janus kinase (JAK), which is phosphorylated when presented with the stimulating myokine (IL-6), which then stimulates the Signal Transducer and Activator of Transcription 3 (STAT3) for phosphorylation and translocation to the cell nucleus to upregulate gene transcription. Then, satellite cell (SC) proliferation and differentiation may occur to enhance skeletal muscle generation, or muscle hypertrophy may occur in the presence of an acute increase in IL-6 concentrations such as during exercise (i.e., skeletal muscle contraction). If high concentrations of chronically elevated IL-6 are present, atrophy of skeletal muscle may occur, thus resulting in sarcopenia, especially in aged skeletal muscle. Figure created using BioRender.

Myogenesis is governed by several signal transduction networks. These varied elements include pathways, signaling, and transcription factors. The MAPK pathways include ERK, JNK, p38 MAPK, and ERK5 pathways [75,76]. These regulators govern multiple steps across the timeline, from activation to maturation. An additional route, PI3K-Akt, is an essential process making the transition from myoblast to mature myocytes. The whole complex process can be illustrated by the fact that growth factors, such as fibroblast growth factor (FGF), hepatocyte growth factor (HGF), and insulin-like growth factor-1 (IGF-1), activate quiescent muscle stem cells, inducing myoblast proliferation by triggering the ERK pathway [75]. Transcription factors are terminal effectors of signaling cascades and produce developmental stage-specific transcripts. Their roles are well established and described in the literature. To cite a few, paired-box protein 7 (Pax7) maintains a population of quiescent satellite cells and, together with myogenic factor 5 (Myf5), plays a role in the expansion of activated myoblasts. Myoblast determination protein (MyoD) is believed to determine the differentiation potential of an activated myoblast and acts together with myogenin and myocyte enhancer factor 2 (MEF2) to drive differentiation. Finally, muscle-specific regulatory factor 4 (MRF4) is required for hypertrophy, although it may have other roles as well [76]. For an example of how one myokine (IL-6) may interact with satellite cells and muscle regeneration, please see Figure 1.

## 7. Potential Myokines Involved in Skeletal Muscle Hypertrophy

Many potential myokines have been implicated in skeletal muscle hypertrophy processes [69]; however, this review will emphasize several that have the potential to influence hypertrophic processes through autocrine mechanisms. According to previous literature and scientific discovery, each myokine chosen for this narrative review is a candidate for influencing the hypertrophic process in skeletal muscle. It is realized that there are many more myokines that may influence hypertrophic processes, but this review is focused on the following with biological, as well as scientific, evidence supporting their putative effects within skeletal muscle hypertrophy processes: apelin, decorin, IL-1ra, IL-6, IL-10, IL-15, irisin, LIF, and a section on the role of other myokines of research interest.

### 7.1. Apelin

It has been demonstrated that there is a decrease in circulating apelin with increasing age, which may be one underlying mechanism whereby skeletal muscle is negatively affected; however, apelin holds promise for restoring skeletal muscle, even in aging. We have demonstrated that 12-week resistance training in younger and older males increases the systemic blood concentrations of apelin, thus suggesting one process whereby skeletal muscle exercise training may enhance skeletal muscle mass [77]. Other research evidence has indicated that apelin concentrations are increased systemically with resistance exercise in resistance-exercise untrained and trained alike [78]. Importantly, Vinel et al. [79] reported that there is an association between apelin, exercise, and skeletal muscle function. In their series of studies, restoring apelin levels with recombinant infusion of apelin indicated that various aspects of skeletal muscle dysfunction (positive anti-inflammatory effect and satellite cell-mediated repair of skeletal muscle regeneration) were restored in aged muscle [79]. Apelin also has a role in restoring skeletal muscle mitochondrial biogenesis, thus increasing energy expenditure through increased vascular mass [79,80]. This restoration of mitochondrial biogenesis has been proposed to enhance the negative effects of sarcopenia in aged skeletal muscle and thus, may promote the anabolic metabolism of this tissue [7]. Further study into apelin and its receptor after a hyper-insulinemic-euglycemic clamp has indicated that in type II diabetic patients and controls, there are no concentration differences found in skeletal muscle tissue biopsies, but the systemic blood-based concentration of apelin was increased in the diabetic cohort [81]. This may be indicative of a compensatory response of apelin to improve insulin sensitivity, glucose metabolism, or promote anabolic action within the skeletal muscle with this disease state. However, these studies predominantly evaluated the systemic (potentially endocrine) effects of apelin. Research done in a mouse model where apelin knockout animals were compared to wild-type found a shift in skeletal muscle fiber type from type II to type I skeletal muscle fibers in response to high intensity interval training in the knockout mice [82]. The research also indicated that the IGF-1 signaling pathway in the apelin knockout model was defective and thus unable to induce hypertrophy, suggesting that apelin is essential in this process [82]. Other research conducted on muscular dystrophy mouse models indicated that apelin was necessary to enhance skeletal muscle satellite cell-mediated repair of skeletal muscle in vivo, thus suggesting the importance of this myokine in promoting skeletal muscle regeneration in a diseased model [83]. Apelin administration also acted as an exercise mimetic in enhancing maternal to fetal improvements in mitochondrial biogenesis in skeletal muscle, which would correspondingly enhance skeletal muscle function and the development of fetal skeletal muscle [84]. Overall, this myokine demonstrates some promise in its ability to exert positive effects on skeletal muscle protein synthesis and may be a potential therapeutic agent to restore skeletal muscle loss with age [79] and, at least in a mouse model, was able to enhance lifespan when systemically administered [85].

### 7.2. Decorin

Decorin competitively binds to and is known to be a negative regulator of myostatin, which indirectly influences and promotes skeletal muscle hypertrophy by decreasing the anti-hypertrophic effects of myostatin [47]. We have shown an acute increase in this myokine systemically with resistance-exercise of various types, suggesting that decorin may have physiological effects on other tissue types as well [86]. Further, in a Zebrafish model, swim training induces hypertrophy in skeletal muscle and correspondingly increases the mRNA of a number of myokines including decorin [87], suggesting an autocrine role for this myokine in skeletal muscle tissue. Transgenic models that overexpress decorin demonstrated an upregulation of anabolic myogenic factors and a downregulation of catabolic myogenic factors [88]. Further research indicates that in a decorin-null mouse model, late-stage tendon healing was impaired, suggesting the necessity of this myokine for skeletal muscle (and tendon) repair after injury [89]. While there is minimal research on this myokine in relation to skeletal muscle, it may have physiological and pathological effects in various tissue types, thus warranting caution in using recombinant forms of it in human research [90,91]. One research trial in human males completing acute resistance-exercise indicated decorin-bound myostatin reduces the activation of the Smad 2/3 catabolic pathway in skeletal muscle tissue biopsies [92]. In a human model with −10% or −20% downhill grade treadmill exercise, which has an exaggerated eccentric muscle action associated with it, systemic decorin expression was significantly higher in the −20% grade trial versus the −10% grade trial [93], which is potentially indicative of a larger anabolic response to eccentric exercise when the eccentric component is more pronounced [94]. As acute exercise has been shown to increase decorin concentrations systemically within physiological limits, it likely acts as a myokine within skeletal muscle and may play a role in affecting different tissue types.

### 7.3. Interleukin-1 Receptor Antagonist (IL-1ra)

IL-1ra is released systemically with acute resistance-exercise and is a well-known anti-inflammatory myokine but may be involved indirectly in ameliorating skeletal muscle loss during times of pathological trauma [95]. Infusion of recombinant IL-1ra during sepsis in a rodent model reduced the loss of skeletal muscle protein and attenuated the inhibition of muscle protein synthesis [96]. Fascinatingly, this occurred in gastrocnemius muscle but not in soleus or cardiac muscle, consequently demonstrating muscle-specific, and potentially fiber-type-specific, effects of this myokine. Further study into this phenomenon in a rat model of sepsis indicated that infusion with IL-1ra was able to attenuate the decrease in insulin-like growth factor-1 levels in gastrocnemius muscle, which may help to preserve skeletal muscle loss during times of trauma [97]. While the evidence for the effectiveness of this myokine is preliminary and predominantly evaluates systemic outcomes, the effects that IL-1ra may have on skeletal muscle protein synthesis warrant further research, especially during catabolic states that negatively affect skeletal muscle.

### 7.4. Interleukin-6 (IL-6)

IL-6 is the quintessential myokine that has multiple roles in skeletal muscle metabolism and may affect multiple tissue types in pleiotropic ways [10]. While IL-6 is a myokine that has been studied extensively in the exercise research literature, relatively little is known about its role in skeletal muscle anabolism and protein synthesis, particularly in humans. It has been speculated that IL-6 has a role in influencing skeletal muscle hypertrophy, but further research will be required to illuminate exactly what that role is. Research in rats has indicated that repeated eccentric resistance-exercise training sessions for 20 days showed increases in muscle size and strength, which correlated with increases in IL-6, phosphorylated signal transducer and activator of transcription 3 (STAT-3), and a decrease in myostatin [98]. Interestingly, skeletal muscle satellite cells are influenced by myokines released during exercise, and this likely has application for maintaining skeletal muscle mass, even into older age [23,99]. Previous research has indicated that IL-6 signaling is responsible for STAT3 activation and is associated with the satellite cell response in skeletal muscle subjected to lengthening (eccentric) contractions [100,101]. IL-6 is also a key myokine which has roles in regulating muscle protein synthesis via activation of the gp130-Akt-mTORC1 axis to enhance contractile protein (actin and myosin) accumulation, ultimately resulting in skeletal muscle hypertrophy [13,102]. Remarkably, rats subjected to 8 weeks of aerobic training at 60% VO_2max_ demonstrated skeletal muscle-specific adaptations where a significant decrease was noted in several myokines in the extensor digitorum longus muscle, including IL-6; however, in the soleus skeletal muscle, IL-6 was the only myokine to remain unchanged in response to the exercise training [103]. This suggests that IL-6 may play another role biologically in certain types of skeletal muscle contractions, but further research will be required to determine what this effect is.

In C2C12 mouse myotubes stimulated to contract electrically, there was an increase in IL-6 secretion, which acted in a paracrine manner to reduce the gene expression of pro-inflammatory mediators TNF-α and IL-1β when co-incubated with mesenchymal stem cells as compared to a control media [104]. However, in a human model where both lean and obese humans were recruited and administered an IL-6 receptor blockade, it was determined that there was no effect on protein turnover at rest, during exercise, or during recovery from exercise [105]. In the same study, a chronic IL-6 receptor blockade increased total and essential amino acid concentrations in only the lean individuals [105]. This suggests the metabolic phenotype of humans will likely influence how myokines may act to influence biological outcomes, an interesting conundrum for researchers. Snijders et al. [106] hypothesized that IL-6 is a key myokine that influences satellite cells in skeletal muscle by inducing the cell-cycle via cMyc and cyclin D1 upregulation after strenuous resistance-exercise, especially if the exercise has an eccentric component to it. While satellite cells move from quiescence to activation to proliferation and fusion/differentiation, there are several growth factors and cytokines/myokines that influence this regulatory cycle. It will be interesting to see what future research will unfold in relation to other myokines of interest in relation to satellite cell-cycle biology.

### 7.5. Interleukin-10 (IL-10)

Research into IL-10 effects on muscle metabolism has determined that it may have a moderating role in skeletal muscle hypertrophy. While IL-10 is known as a predominantly anti-inflammatory myokine, the effects it has on macrophage phenotype switching may be key to understanding its role in the normal growth and regeneration of skeletal muscle [31]. IL-10 was involved in M1 to M2 macrophage phenotype switching in a mouse model where a contusion injury was applied to skeletal muscle [31,107]. An in vitro myoblast study determined that IL-10 suppressed exogenously administered IL-1β inhibition of insulin-like growth factor-1 (IGF-1) and promoted myogenin (a key regulator of skeletal muscle regeneration) and myosin heavy chain accumulation [108]. Fascinatingly, mouse models lacking IL-10 challenged with lipopolysaccharide (LPS) found that the inflammatory response in skeletal muscle was greater in that IL-6 was elevated for a longer time period than in the models still maintaining IL-10 [109,110]. This suggests a fine balancing act between the timing of when various types of myokines are required for the growth and regeneration of skeletal muscle fibers after damage. Further, it points to the fine-tuning that the homeostatic mechanisms play in ensuring the recovery and regeneration of skeletal muscle after insult. More research into the effects that IL-10 has on the hypertrophy of skeletal muscle is warranted.

### 7.6. Interleukin-15 (IL-15)

Over 20 years ago, an initial study on the effects of IL-15 on mouse skeletal muscle myogenic cells overexpressing this myokine determined that there was a 5-fold increase in sarcomeric myosin heavy chain and actin in differentiated myotubes [111]. The authors suggested that IL-15 pathway modulation may maintain skeletal muscle during times of muscle loss such as cachexia [111]. Further research demonstrated varying effects of the IL-15 myokine in stimulating myosin heavy chain accretion after myoblast differentiation [112], attenuating the proteolytic rate in rat skeletal muscle [113] and enhancing mRNA in skeletal muscle of aged rats and quail in response to atrophic stimuli [114]. This research led to the conclusion that IL-15 demonstrates anabolic effects both in vitro and in vivo in 2007 [115]. Research continued in this manner and determined that, following a denervation injury of skeletal muscle in a Sprague–Dawley rat model, IL-15-protein kinase B (Akt) signaling promoted cross-talk between T-cells and skeletal muscle to decrease muscle cell damage and inflammation, potentially promoting a greater pro-anabolic environment [116]. Recent human evidence indicates that the IL-15/IL-15Rα pathway was stimulated after acute resistance-exercise and correlated with the myofibrillar fractional synthetic rate in skeletal muscle, suggesting the potential influence this myokine has on anabolism [117]. More research on IL-15 will reveal the intricacies of this myokine in skeletal muscle and whether it is anti-catabolic or pro-anabolic in nature.

### 7.7. Irisin

Irisin is known to have multiple effects on skeletal muscle metabolism, the most prevalent being the “browning” of white adipose tissue [118], and may act as an exercise mimetic for skeletal muscle hypertrophy [119]. Initially, it was found that irisin was able to upregulate IGF-1 and downregulate myostatin in vitro, suggesting a role in skeletal muscle anabolism [120]. Corroborating this finding, histological analysis of tilapia demonstrated irisin injection was able to induce skeletal muscle growth by enhancing mRNA for IGF-1 and decreasing mRNA for myostatin [121]. Further research indicated that recombinant irisin injection in a mouse model induced hypertrophy and strength gains in uninjured skeletal muscle while at the same time increasing pro-myogenic genes via IL-6 activation [122]. In addition to this finding, cultured bovine satellite cells had an increase in mRNA for FNDC5, along with IL-6, IL-15, and BDNF, in the differentiated versus undifferentiated state, thus hinting at roles for these myokines in the sequential events of satellite cells in muscle regeneration [123]. Additionally, irisin injection or infusion in a Sprague–Dawley rat model increased AKT1 and PI3K gene expression in harvested skeletal muscle tissue, suggesting a role for this myokine in the autocrine regulation of skeletal muscle mass via the Akt/PI3K/mTOR pathway [124]. However, a study completed 5 years ago concluded that there were limited effects of irisin in relation to skeletal muscle anabolism in a rat hindlimb model [39]. Our recent research evaluating myokine responses to a 12-week resistance-exercise training program in younger and older adult males demonstrated that irisin was lower in the aged males following the training [77]. Thus, it seems that irisin may play a role in skeletal muscle, but further research will be needed to explain exactly what its part may be in relation to anabolism and catabolism.

### 7.8. Leukemia Inhibitory Factor (LIF)

LIF has shown some potential in enhancing anabolic action in skeletal muscle. Initial research on this myokine indicated that if LIF was administered after crush injury, the injured muscle had an increased rate of regeneration [125]. Further data in relation to this myokine indicates that LIF promotes survival in skeletal muscle regeneration and myogenesis [126]. LIF was also able to activate gp130-Akt signaling and stimulate protein synthesis via the mTORC1 pathway in cultured myotubes [102]. This preliminary evidence for LIF indicates a potential role it may play in enhancing anabolic action in skeletal muscle; however, human research on this myokine is desperately needed to determine its effects in human physiology.

### 7.9. Other Myokines

There is a wide array of other myokines that hold promises for future research discoveries in relation to skeletal muscle anabolism. Briefly, a few other potential myokines with potential anabolic effects will be discussed. First, angiopoietin-1 (ANGPT-1), a well-known angiogenic factor, may also have a role in cross-talk with skeletal muscle. Within an in vitro experiment, ANGPT-1 was able to enhance myogenic regulatory factors MyoD and myogenin, which led to an increase in myosin heavy chain accumulation [127]. Although this data is preliminary, further data from a human-based study examining heat therapy after eccentric resistance-exercise determined that ANGPT-1 was increased and was able to improve muscle recovery and repair following the muscle damaging bout of exercise [128]. Secondly, interleukin-4 (IL-4) may hold capability for enhancing skeletal muscle hypertrophy by recruiting myoblasts to fuse into myotubes to enhance growth, at least in cultured skeletal muscle cells [129]. Further evidence suggests that IL-4 and its receptor’s mRNA and protein content are enhanced in human skeletal muscle biopsies after 6 weeks of high-intensity resistance-exercise training [130]. The authors of the aforementioned study speculate that IL-4 is involved in skeletal muscle hypertrophy and has anti-inflammatory effects that would aid in skeletal muscle damage control following strenuous resistance-exercise [130]. Thirdly, interleukin-7 (IL-7) is speculated to have a role in anabolism, as it is both expressed and secreted by skeletal muscle in vitro and in vivo [131]; however, further direct evidence linking IL-7 to skeletal muscle hypertrophy and anabolism is required to confirm these initial results. In a mouse model, secreted protein acidic and rich in protein (SPARC) is considered a myokine [14] that may influence the cytoskeleton structure of skeletal muscle by interacting with actin to maintain force production when skeletal muscle is challenged with a fatiguing protocol [132]. Further evidence linked IGF-1 signaling with increased SPARC intramuscularly in an in vivo mouse model [133]; however, other evidence in a Sprague–Dawley rat model observed a compensatory response of SPARC to hindlimb immobilization, thus suggesting a role in enhancing anabolism in response to the stressor [134].

Other myokines may be involved in satellite cell activation within skeletal muscle [23]. Brain-derived neurotrophic factor (BDNF) is a myokine potentially influencing skeletal muscle via its expression from satellite cells [135]. An in vivo mouse model where BDNF was knocked out of skeletal muscle specifically demonstrated that skeletal muscle regeneration was compromised while satellite cell regulation was also negatively affected [135]. These results indicate the potential importance of BDNF in promoting regenerative capacity in skeletal muscle satellite cells. Bone morphogenetic proteins (BMPs) are groups of proteins that have a myokine role via signaling through the TGF-β pathway [136]. An isotype of this group of proteins (BMP7) positively influences skeletal muscle via mTOR signaling, promoting muscle growth and inhibiting skeletal muscle atrophy [136,137]. Fibroblast growth factors (FGFs) are a group of signaling molecules that regulate many different cell processes [138]. Interestingly, in a mouse model, FGF-19 was able to increase muscle fiber size and attenuate muscle atrophy via stimulation of the S6K1 pathway, which may have clinical benefits in those with cachexia and sarcopenia [139]. Granulocyte colony-stimulating factor (G-CSF) has involvement in preserving a niche of satellite cells (Pax7Hi) in aged skeletal muscle satellite cells, potentially preserving skeletal muscle into older age [140]. Further, hepatocyte growth factor (HGF) has been shown to be present in skeletal muscle [141,142] and to activate quiescent satellite cells [141,143]. It may not lead to skeletal muscle regeneration, as differentiation is inhibited both in vitro and in vivo [143], but it may lead to the activation of the AKT/mTOR/S6K-1 pathway with exogenous administration of the myokine [144]. The above-mentioned conflicting results point to the need for more research on HGF, as it is an important growth factor that is also considered a myokine.

## 8. Future Research Directions and Challenges

While the role of myokines in skeletal muscle anabolism and hypertrophy is somewhat elucidated in cell culture and animal models, much more research on myokine roles in skeletal muscle of humans is urgently needed. It is thought that there may be over 600 myokines according to some estimates [8], but it is highly likely there is a plethora more of these bioactive compounds that have yet to be discovered. The challenge moving forward in this area of research is the functional role each of these myokines have in the physiological milieu and in maintaining homeostasis. Likely, there is redundancy in the multiple myokines that are released. Research is only beginning to understand the role of myokines in human skeletal muscle, and many more questions need to be answered in relation to this interesting topic [6]. Aging and resistance-exercise training modify some but not all resting concentrations of certain myokines [77]. Understanding how to modify potentially advantageous myokine responses to exercise training and other interventions will aid in reducing the risk of developing sarcopenia with age. Further exploration of this topic is warranted, and future research should focus on the underlying mechanisms whereby myokines have a positive or negative influence on skeletal muscle. Future mechanistic work should identify whether other myokines besides myostatin influence the inhibition of catabolic pathways in skeletal muscle, whether certain myokines can directly stimulate muscle protein synthesis and the mTOR pathway, and if other myokines besides IL-6 and LIF influence the satellite cell cycle. Furthermore, there are unknowns around how the modified myokine/cytokine milieu within the aging context will influence anabolic and catabolic pathways and whether the changes observed with aging are modifiable with various interventions. Understanding why and how myokines respond to exercise or other stressors is the key to determining skeletal muscle’s role in maintaining homeostasis. Further, this understanding needs to be determined across the lifespan, particularly in older adults, as there is a high incidence of skeletal muscle loss with age. By determining the role of myokines in promoting anabolism and reducing catabolism, researchers will be better able to address the pressing health need of sarcopenia in our aging population.

## 9. Conclusions

In summary, there are many potential myokines that have an influence on skeletal muscle. There are also many more myokines that likely influence distal tissues after their release from skeletal muscle. This review summarizes a few of the potential myokines that have a putative effect on skeletal muscle anabolism from an autocrine perspective. More research efforts in this area are required to further elucidate a mechanistic understanding of the underlying manner by which myokines influence skeletal muscle. Specific areas of focus for future research include the potential cross-talk between inflammatory pathways and anabolic pathways in affecting skeletal muscle hypertrophy, whether there are skeletal muscle fiber-type specific alterations in certain types of myokines, and whether different types of muscle actions (i.e., concentric, eccentric, isometric, voluntary, involuntary) all influence myokine production and the maintenance of skeletal muscle homeostasis in a similar manner. Finally, the goal of this research is to decrease the risk of developing sarcopenia in older age and to preserve functional independence through maintenance of skeletal muscle homeostasis across the lifespan.

## Figures and Tables

**Table 1:** Myokines and their autocrine action.

Myokine	Autocrine Action	Key Evidence
Apelin	Activates an AMPK-dependent mitochondria biogenesis; it promotes autophagy and decreases inflammation [44].	Increased the weight of studied muscles in both aged wild-type and *Apln*^−/−^ mice, which was associated with fiber hypertrophy [45].
Decorin	Binds and blocks myostatin, reducing inhibitory effect on growth; sustains hypertrophy after resistance exercise [46].	Mouse and humans increased expression of genes involved in pathways of skeletal muscle growth after Decorin’s overexpression. Rising plasma levels in response to resistance exercise; increases muscle hypertrophy [47].
IL-1ra	IL-1R1-deficient mice develop insulin resistance faster than their wild-type littermates do [48]. Platelet-rich plasma and IL-1β antagonist receptor peptide attenuate the inflammatory process of muscle injury in Wistar rats [49].	Amplified glucose-stimulated insulin secretion from human islets correlated with donor BMI. Islets from obese donors were sensitized to the insulinotropic effects of this cytokine, whereas the stimulatory effects of IL-1β were lost in islets from obese T2D patients [50].
IL-6	Activates AMPK, amplifies GLUT4 translocation and glucose uptake, augments IL-10 and IL-1ra [13]. Activation of satellite cells and increased fatty acid oxidation [22].	Hypoxia could improve muscle hypertrophic response following resistance exercise through IL-6/STAT3-dependent myogenesis and immune cells-dependent muscle regeneration [51].
IL-10	Induces M2 phenotype through STAT3 signaling during macrophage polarization [30]. Suppresses proinflammatory gene expression [52].	Co-culturing muscle cells with macrophages activated with IL-10 to the M2 phenotype increased myoblast proliferation [31].
IL-15	Activates PPAR-delta-enhancing muscle oxidative metabolism; augments fatty acid oxidation, nucleotide metabolism [46].	Hypertrophies phenotype in myotubes and increased contractile protein accumulation in skeletal myogenic cultures (mouse, human, and bovine) [53,54].
Irisin	AMPK activation, which triggers p38 MAPK signaling and GLUT4 vesicle trafficking to the plasma membrane [55]. Proliferation of murine myoblasts. Increased number of activated myoblast nuclei [22].	In vitro, it stimulates glucose uptake via AMPKα2-mediated p38 MAPK activation in muscle cells and by examining GLUT4 translocation [34].
LIF	Increases glucose uptake; it is involved in hypertrophy modifications [46]. Enhances satellite cell proliferation [56]. Accelerated proliferation of satellite cells; skeletal muscle regeneration [22].	Supply of LIF for 21 days to LIF (−/−) mouse restored muscle hypertrophy [57]. Levels of LIF increased nine-fold after a bout of resistance exercise (humans) [56].
Myostatin	Negative regulation of muscle mass by activating activin, which phosphorylates SMAD2/3. SMAD 2/3 can inhibit the transcription factor JunB, which normally promotes muscle growth and inhibits atrophy by blocking FoxO3. Evidence suggests that conjugated action affects Akt/mTOR axis [21,22].	JunB inhibits the activation of proteolysis by FoxO3 in C2C12 myotubes (from mouse) [58]. Inhibition of myostatin and activins resulted in a hypertrophic response, with muscle mass increasing >150% in mouse models of muscular dystrophy [59].
BDNF	Its deficiency at the cell level leads to reduced fatty acid-induced mitofission and mitophagy [22]. Involved in the activation of AMPK and the promotion of lipid oxidation within the muscle [60].	Mice have exacerbated body weight gain, increased intramyocellular lipid deposition, reduced energy expenditure, poor metabolic flexibility, and more insulin resistance [36].

Note: Some of the main myokines with their attributed autocrine action and corresponding main evidence. Interleukin-1 receptor antagonist (IL-1ra); interleukin-6 (IL-6); interleukin-10 (IL-10); interleukin-15 (IL-15); leukemia inhibitory factor (LIF); brain-derived neurotrophic factor (BDNF).

## Data Availability

No new data were created or analyzed in this study. Data sharing is not applicable to this article.
